# Hypophosphites in Phthisis

**Published:** 1860-05

**Authors:** 


*Hypophosphites in Phthisis.*—In the *Medical Press* for Jan. 14th, is published a letter from Dr. J. J. Campbell, of Brooklyn, to Dr. J. Winchester, reporting the effects of the hypophosphites of lime and soda in his own case. The night-sweats soon ceased to trouble, and the nervous system so improved as to permit sound and refreshing sleep. He says "When I commenced the use of this remedy, five weeks ago, I weighed only 147 lbs.; now I weigh 161 lbs., a slight increase over my usual weight. My appetite is good, I sleep well, and I feel as if I were going to live in spite of the formation of a cavity in the upper portion of my right lung." This is certainly an encouraging result of the new remedy. The hypophosphites are much more pleasant to take than cod-liver oil, and we hope the effects following its use may be a still more important improvement.—*American Medical Monthly.*

